# A review of toolkits and case definitions for detecting enteric fever outbreaks in Asian and African countries from 1965-2019

**DOI:** 10.7189/jogh.11.04031

**Published:** 2021-05-29

**Authors:** Asif Khaliq, Mohammad Tahir Yousafzai, Salman Haq, Rahima Yaseen, Sonia Qureshi, Fahad Rind, Zahra A Padhani, Ayub Khan, Abdul Momin Kazi, Farah Naz Qamar

**Affiliations:** 1 Department of Pediatrics and Child Health, The Aga Khan University Hospital, Aga Khan University Karachi, Pakistan; 2 Kirby Institute, University of New South Wales, Australia; 3 Ziauddin Medical College, Ziauddin University Karachi, Pakistan; 4 School of Public Health and Social Work, Queensland University of Technology, Brisbane, Australia; 5 Institute of Global Health and Development, Aga Khan University Hospital, Karachi

## Abstract

**Background:**

This review assessed the case definitions, diagnostic criteria, antimicrobial resistance, and methods used for enteric fever outbreaks and utilization of any unified outbreak score or checklist for early identification and response in Asia and Africa from 1965-2019.

**Methods:**

We searched enteric fever outbreaks using PubMed, Google Scholar, and the Cochrane library. Studies describing a single outbreak event of enteric fever in Asia and Africa from 1965-2019 were reviewed. We excluded case reports, letter to editors, studies reporting typhoid in conjunction with other diseases, the Centers for Disease Control and Prevention (CDC) trip reports, the World Health Organization (WHO) bulletins report, data from mathematical modeling and simulation studies, reviews and ProMed alert. Also, non-typhoidal salmonella outbreaks were excluded.

**Results:**

A total of 5063 articles were identified using the key terms and 68 studies were selected for data extraction. Most (48, 71%) outbreaks were from Asian countries, 20 (29%) were reported from Africa. Only 15 studies reported the case definition used for case identification during an outbreak and 8 of those were from Asia. A third (20, 29%) of the studies described antibiotic resistance pattern. 43 (63%) studies contained information regarding the source of the outbreak. Outcomes (hospitalization and deaths) were reported in a quarter of studies. Only 23 (29%) of the studies reported outbreak control strategies while none reported any unified outbreak score or a checklist to identify the outbreak.

**Conclusion:**

This review highlights the variability in detection and reporting methods for enteric fever outbreaks in Asia and Africa. No standardized case definitions or laboratory methods were reported. Only a few studies reported strategies for outbreak control. There is a need for the development of a unified outbreak score or a checklist to identify and report enteric fever outbreaks globally.

Enteric fever is a major public health concern especially in low middle-income countries (LMICs) [1]. The disease is caused by subspecies *enterica serovar Typhi (*S. *typhi) and Paratyphi (*S. *paratyphi). Typhi* is responsible for causing typhoid fever, while Enteric fever is a group of enteric infection caused by *Salmonella enterica.* The *Salmonella enterica* has different subspecies serovars, which are responsible to cause varying types of enteric infections, such as typhoid fever by *S. typhi* and paratyphoid fever by *S. paratyphi* A, B, and C [2,3]. The disease is characterized by high-grade fever, fatigue, malaise, headache, and certain gastrointestinal symptoms including abdominal pain, diarrhea, or constipation [3,4]. The disease is highly endemic in most of the LMICs. Compared with industrialized nations, people living in Asian and African countries are vulnerable to enteric fever. Despite having high endemicity of enteric fever, the disease cannot be diagnosed clinically from the clinical sign and symptoms [5].

The symptoms of enteric fever are non-specific and are closely relate to other febrile illnesses, such as malaria, dengue, and influenza [5]. Clinicians preferred to diagnose enteric fever with serological and other laboratory tests for diagnosing enteric fever. The Widal test, Typhi dot and Tubex-M test are the commonly prescribed serological tests for the initial diagnosis of enteric fever [6]. However, for the confirmatory diagnosis of enteric fever, blood, bone marrow or other body specimen is recommended. Among different body specimen, the bone marrow culture has the highest sensitivity of 96% for Salmonella species. Despite bone marrow culture highest sensitivity, blood culture is considered as the gold standard for the confirmatory diagnosis of enteric fever, because it is less invasive compared with bone marrow culture. The culture sensitivity of blood culture decreased to around 30% from 60% in patients having less than a week symptoms history and in patients with a history of antimicrobial use [7-10]. The rising antimicrobial resistance against *S. typhi* is a global threat. The antimicrobial agents, such as amoxicillin, chloramphenicol and co-trimoxazole served as the first line of choice for treating enteric fever, however, globally more than a third of the population is resistant to these agents [1,11]. Enteric fever patients who are resistant to amoxicillin, chloramphenicol and co-trimoxazole are found to have Multidrug Resistance (MDR) enteric fever [12]. The emergence of MDR enteric fever in the late 1980s and the early 1990s has shifted the therapeutic choice to second-generation fluoroquinolones and third-generation cephalosporins [13,14]. Still, the issues related to therapeutic failure reported due to the emergence of Fluoroquinolones resistance (FQR) and Extensive Drug Resistance (XDR) [11,14,15]. The XDR enteric fever was first observed in November 2016 from the southern parts of Pakistan. The therapeutic management of XDR enteric fever seems like a medical challenge for the health practitioners, because of the limited choice of antibiotics. In general, the macrolides (Azithromycin) and carbapenems (Meropenem or Imipenem) are recommended for treating XDR enteric fever cases [16-18].

The global burden of enteric fever is not homogenous. Most of the Asian and African countries are highly endemic to enteric fever. The incidence rate of enteric fever in most of the Asian and African countries ranged from 100 to 700 per 100 000 population, while the incidence rate of enteric fever in other regions is less than 15 per 100 000 [3]. Factors, such as living in urban slums, inaccessibility to safe drinking water, restaurant or cafeteria food, close contact with enteric fever patients and prior use of antibiotics significantly increased the risk of enteric fever [18,19]. People living in Asian, African, and Latin American countries are deprived of safe drinking water, adequate sanitation, and proper shelter, which in turn are responsible for many communicable and infectious diseases including enteric fever [19,20]. In this regard, it is essential to know about the case definitions, diagnostic criteria, antimicrobial resistance, methods used for outbreaks control and use of any unified outbreak scoring or a checklist for the identification of enteric fever outbreaks in Asia and Africa from 1965-2019.

## METHODS

We searched PubMed, Google Scholar and the Cochrane library using a combination of Medical Subjects Heading (MeSH) and Keywords, i.e, “Salmonella Infections” [Mesh] OR “Typhoid Fever” [Mesh] OR “Paratyphoid fever” OR “Enteric fever” AND “Disease Outbreaks”[Mesh] OR outbreak*. In addition, filters for the publication year from 1965 to May 25, 2019, English language and human studies were used (where applicable) (Supplementary file 1). All studies indicating documentation of clinical features, based on title and/or abstract, were retrieved in the full text where available.

We conducted a parallel search to examine the included studies reference lists and pertinent systematic reviews for additional studies.

### Eligibility criteria

We considered original research articles that described at least one outbreak event of enteric fever in humans from 1965 to May 2019. We included all those studies that described enteric fever (typhoid or paratyphoid) outbreak event in Asia and Africa irrespective of age, gender, institutional setting, treatment method and source of transmission. Studies with enteric fever cases identified based on clinical criteria alone or confirmed by culture (blood, bone marrow, stool, or any other sterile fluid) or serology (Widal test/Typhi Dot test), were included. We excluded case reports studies using a clinical diagnosis only, letter to editors, studies reporting typhoid in conjunction with other diseases, CDC trip reports, WHO bulletins report, data from mathematical modeling and simulation studies, reviews and ProMed alerts (**Table 1**).

**Table 1:** List of exclusion criteria

Case-reports
Editorial and letters to the editor
Newspaper reports
Book chapters
Non-human studies
Not published in the English language
Reports or bulletin of WHO, CDC and ProMed alerts
Enteric fever outbreaks before 1965
Studies showing no evidence of enteric fever outbreak
outbreaks due to Non-typhoid Salmonella
Enteric fever outbreaks outside –Asia and Africa
other diseases discussed along with enteric fever outbreak
Mathematical Modeling and simulation studies
Outbreaks from the same hospital/region and during the same period were considered once
Same hospital/region and during the same period were considered
Narrative reviews
Systematic reviews

Studies published from the same hospital/region and during the same time were considered as duplicate/overlapping data and counted once.

### Screening process

**Figure 1:**
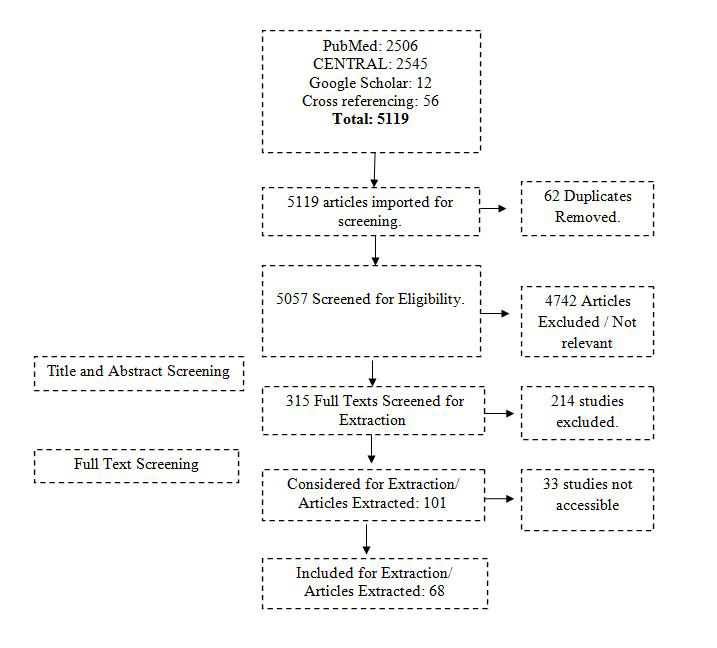
PRISMA flow diagram.

All papers searched through different databases were imported into the endnote library. Duplicate records were removed. Two co-authors (AK and FR) independently initiated the screening of the papers. In the first step, titles and abstracts were independently screened and ineligible records were excluded. At the end of the first phase of screening, both reviewers met and discussed the papers included and excluded. The third reviewer (ZF) resolved any disagreements between the two independent reviewers. The second screening was based on a full-text review of the remaining papers. Again, AK and FR reviewed the full text of all papers and any paper not fulfilling the eligibility criteria was excluded. Disagreements between the two reviewers were resolved by the third reviewer (ZF). The remaining papers after the second screening were used for data extraction. The whole screening process with the number of records is provided in the PRISMA flowchart (**Figure 1**).

### Data extraction, management, and analysis

We created an excel sheet for data extraction. The excel sheet included variables such as first author name, year of publication, country of publication, study design, the case definition for enteric fever, number of cases based on culture confirmation, serological testing and/or clinical suspected, diagnostic methodology used for identifying the outbreak, tool or a unified score system to identify the outbreak, source of the outbreak (water or food item reported as the source for transmitting the outbreak), number of hospitalizations (number of studies reporting any hospitalization and the proportion of affected people hospitalized for typhoid during the outbreak), antimicrobial resistance or sensitivity, control measures, the effectiveness of the control measures or strategies in containing the outbreak. Data extraction was performed by the two co-authors (SH and RY) and any disagreements in data extraction were addressed by the third co-author SQ.

Extracted data were classified into studies based on reported outcomes, age distribution, and geographical regions and by the outbreak periods. Frequency tables by country and region for each outcome variable were tabulated in Microsoft Excel latest version (Microsoft Inc, Seattle, WA, USA).

## RESULTS

A total of 5063 studies were extracted (2506 from PubMed, 2545 from Cochrane library and 12 studies were from Google Scholar). Additional 56 records were identified through cross-referencing, making the total records N = 5119. There were 62 duplicates, which were removed, and the remaining 5057 records were used for screening. The selection process is illustrated in the PRISMA study flow diagram (**Figure 1**). A total of 68 studies were included for the final review.

55 studies were selected based on more than one inclusion criteria.

### Geographical Distribution of Enteric Fever Outbreak in Asia and Africa

**Figure 2:**
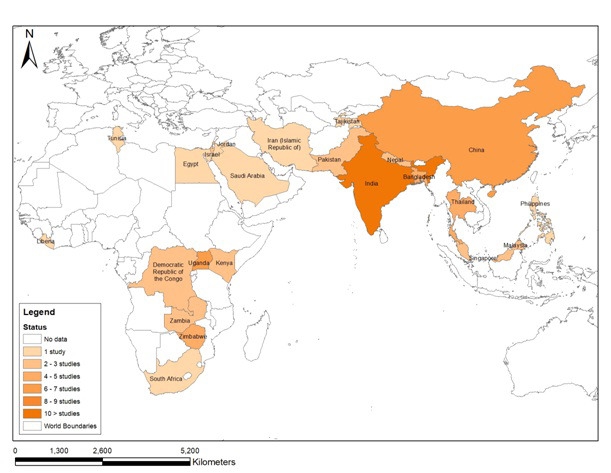
Geographical distribution of Enteric fever outbreaks in different countries of Asia and Africa.

Of the 68 selected studies, 48 were from Asia and 20 from Africa. Spatial distribution of the outbreaks is presented in the geospatial map (**Figure 2**).

The first outbreak of enteric fever from Asia was reported from India in 1975 while in Africa, it was reported from South Africa in 1978. From 1976 to 1986, 5 studies regarding outbreaks were identified, 3 from Asia and 2 from Africa. The number of outbreak studies retrieved increased continuously in the succeeding decades ie, 17, 19 and 26 in the periods1987-97, 1998-08, and 2009-19 respectively.

### Enteric fever case definitions

There were 15 studies, which reported a case definition of enteric fever for the identification of cases in the outbreak. Among these 15 studies, 8 (53%) studies were from Asia and 7 (47%) from Africa.

Presence of fever along gastrointestinal disorders (ie, abdominal discomfort, diarrhea, constipation, nausea, vomiting) as case definition criteria for enteric fever was used in seven papers from 6/7 (86%) from Africa and 1/8 (12.5%) from Asia. Fever exceeding 37.5°C or 38.0°C for more than 3 days was used as a case definition in two studies. In six studies fever greater than 38°C was either alone or along with headache. The various case definitions used are presented in **Table 2**.

**Table 2:** Clinical signs used for defining a case of enteric fever in an outbreak (n = 15)

Region	Country name	Year of outbreak	Clinical or suspected symptoms											
**Fever (n = 13)**	**Abdominal pain/cramps (n = 10)**	**Diarrhea (n = 10)**	**Vomiting (n = 8)**	**Nausea (n = 3)**	**Constipation (n = 6)**	**Headache (n = 7)**	**Weakness/fatigue (n = 5)**	**Malaise (n = 4)**	**Cough (n = 4)**	**Intestinal Perforation (n = 32)**	**Others (n = 4)**
Uganda	2009	*	*		†			†	†			†	†
**Africa**	Uganda	2009-2011	*	*					†	†			†	†
	Zimbabwe	2011-2012	*	†	†			†	*		*	*		
	Zimbabwe	2012	*	†	†			†	*		*	*		*
	Zimbabwe	2012	*		†	†		†	†		*	*		
	Uganda	2015	*	†	†	†	†	†	†	†				†
	Zambia	2017	*	*	†	†		†		†				
**Asia**	Israel	1985	*											
	Jordan	1989	*		†	†								
	Myanmar	2000	*	†	†			†						
	Nepal	2002-2004	*											
	Pakistan	2004	*	*	*	*				*				
	Singapore	2007	†	†	*	†	†							
	Singapore	2007		†	*	†	†							
	China	2010	*						*					

*Studies support that the sign must be present.

†Studies support that the sign may be present along with other symptoms.

2-3 African studies used the presence of intestinal perforation, negative malarial parasite test and failure to respond to anti-malarial treatment as a definition of enteric fever. **Table 1** shows the details of clinical signs used for defining a case of enteric fever in an outbreak setting. None of the studies reported the use of any unified scoring or a tool for the identification of enteric fever outbreak.

### Enteric fever laboratory diagnostic methods

Only 25 studies provided information regarding enteric fever laboratory confirmation. Out of which, 17/25 (68%) studies were from Asia and 9/25 (36%) were of African origin. The definition for a laboratory-confirmed case of enteric fever was presented by only 3/25 (12%) studies, where a laboratory-confirmed case was defined as the *presence of S. typhi or S. paratyphi* in the blood (n = 3), stool (n = 3) or any other body fluids cultures urine (n = 2) and bone-marrow (n = 1). However, there were 4/25 (16%) studies in which screening test, such as, Widal test and Tubex TF were performed. The screening test performed in Asian countries was the Widal test, whereas in African studies Tubex TF was commonly advised. Out of these four studies, only one presented probable case definition of enteric fever based on a positive serological test (Widal’s test, typhidot, tubex). There were 20/25 (80%) studies in which blood culture ordered for the confirmation of enteric fever (**Table 3**).

**Table 3:** Screening and diagnostic test for enteric fever investigation (n = 25)

Region	Country name	Year of outbreak	Screening test		Laboratory confirmed test			
**Widal test (n = 3)**	**Tubex TF test (n = 1)**	**Blood culture (n = 20)**	**Stool culture (n = 12)**	**Urine culture (n = 5)**	**Others (n = 3)**
**Africa (n = 8)**	Tunisia	2004-05			*	*		
	Zimbabwe	2007			*	*	*	
	Uganda	2009		*	*			
	Uganda	2009			*	*		
	Zambia	2010-12			*	*		
	Zimbabwe	2011			*	*	*	
	Zimbabwe	2012			*			
	Zambia	2017				*		
**Asia (n = 17)**	Singapore	1979			*	*	*	
	Israel	1985			*			
	Pakistan	1988			*			
	Bangladesh	1989-90	*		*			†
	India	1990			*			
	India	1997			*			
	India	2000	*		*			
	Nepal	2002			*			
	China	2004						‡
	Singapore	2007				*		
	Singapore	2007				*		
	China	2010			*	*		
	India	2010			*	*	*	§
	Myanmar	2010			*			
	Malaysia	2012				*		
	India	2013			*			
	India	2014	*		*			

†Bone marrow.

‡Gall bladder fluid.

§Rectal swab.

### Antimicrobial resistance of enteric fever

20 studies, 15(75%) from Asia and 5 (25%) from Africa reported the antimicrobial sensitivity pattern of the isolates identified in the outbreak. 15 (75%) studies reported MDR isolates, 4 (20%) reported FQR, and one (5%) study reported XDR *S. typhi* isolate during the outbreak.

In the Asian region, the first reported case of MDR was observed in Pakistan in 1988. Later, a series of MDR isolates were reported from other Asian countries: Bangladesh in 1989, India in 1990, Iran in 1992, Tajikistan in 1997, Thailand in 1999, Nepal in 2002 and China in 2006. In African countries, MDR was first observed in 2004 in Kenya and later in Uganda in 2011. The cases of FQR were first reported from Tajikistan in 1997, followed reports from Kenya and Nepal also. (**Table 4**).

**Table 4:** Enteric fever drug resistance (N = 20)

Regional division	Country	Year of outbreak	MDR (n = 15)	FQR (n = 4)	XDR (n = 0)	Other (n = 4)
**Africa (n = 5)**	Kenya	2004	*			††
	Tunisia	2004	–	–	–	–
	Uganda	2009	–	–	–	–
	Uganda	2011	*			
	Kenya	2014		‡		
**Asia (n = 15)**	Pakistan	1988	*			
	Bangladesh	1989	*			
	Bangladesh	1990	†			
	Bangladesh	1990	*			
	India	1990	*			
	Bangladesh	1992	*			
	Iran	1992	*			
	Tajikistan	1997	*	§		‖
	Thailand	1999	*			‖
	India	2000	*			¶
	Nepal	2002	*	*		
	China	2006	*			
	Nepal	2009		§		
	Thailand	2009	–	–	–	–
	India	2010	*			

MDR – multidrug resistance, FDR – fluoroquinolone drug resistance, XDR – extensive drug resistance

†Strains were resistant to chloramphenicol.

‡Resistance to ciprofloxacin 54% in tested strains.

§Strains were resistant to nalidisic acid but sensitive to floroquinolones.

‖Strains were resistant to streptomycin and tetracycline.

¶Strains were resistant to tetracycline.

### Enteric fever outbreak source investigation

Out of 68 selected studies, a total of 41 (60%) studies contained information regarding the source of enteric fever outbreak. Among these 41 studies, 33 (80%) were of Asian origin while 8 (20%) were from Africa. In addition, there were 11 (25%) studies, in which more than one source of the outbreak was reported. The sources of outbreaks were broadly categorized into 4 categories: water contamination, food contamination, carrier transmission and inappropriate sanitation & hygiene. **Table 4** shows the sources of the outbreaks by country and regions.

Inappropriate sanitation and water contamination were identified as the major cause of enteric fever outbreaks in both Asian and African countries. There were 20 (50%) and 17 (41%) studies in which inappropriate sanitation and fecal contamination of drinking water were identified as the leading cause for the spread of the enteric fever. Similarly, there were 12 (29%) studies in which food contamination was identified as other associated cause for enteric fever outbreak transmission. Different food items responsible for the spread of enteric fever outbreak were cowpea salad, coconut milk, cream cake, dessert buns, fish, mashed potatoes, meat, mousse cake, noodles, oysters, raw chicken, raw pork, raw vegetables, rice, and sweets. The food-borne enteric fever outbreaks were only reported from Asian countries and were observed in China, India, Jordan, Singapore, Saudi Arabia, and India. There were 6 (15%) studies that testified transmission of enteric fever from person to person, ie, close contact with Typhi positive cases, food caterer or cook (**Table 5**).

**Table 5:** Reporting source of enteric fever outbreak by country and region (n = 41)

Regional division	Country name	Year of outbreak	Source of outbreak			
**Drinking water contamination (n = 17)**	**Food contamination (n = 12)**	**Transmission through carriers (n = 6)**	**Inappropriate sanitation and hygiene (n = 20)**
**Africa (n = 8)**	South Africa	1978			*	
	Uganda	2009	*			
	Democratic Republic of Congo (DRC)	2011	*			*
	Zimbabwe	2011	*			*
	Zimbabwe	2012				*
	Kenya	2014				*
	Uganda	2015	*			
	Zambia	2017	*			*
**Asia (n = 33)**	India	1975				*
	Singapore	1979		*		
	Israel	1986				*
	Pakistan	1988	*			
	Saudi Arabia	1988		*		
	Jordan	1989		*		
	India	1991				*
	Thailand	1991				*
	Pakistan	1992			*	*
	India	1995				*
	Singapore	1996		*		
	India	1997	*			
	Tajikistan	1997	*	*		*
	Thailand	1999				*
	Myanmar	2000	*		*	*
	China	2004	*		*	*
	China	2004	*			
	Pakistan	2004	*			
	India	2005				*
	China	2006	*			
	India	2007				*
	Singapore	2007		*		
	Singapore	2007		*		
	Philippines	2008	*			
	Malaysia	2009				*
	Nepal	2009			*	
	China	2010	*	*		
	China	2010		*		
	Malaysia	2012				*
	China	2013		*		
	India	2013	*	*	*	
	India	2014	*			*
	China	2015		*		

### Preventive measures for the control of enteric fever

The information regarding different preventive strategies for controlling enteric fever outbreak was presented by 15 (36%) studies. The different preventive strategies used as a response to the outbreak were health education, mass immunization, building and construction of sewerage plant and pipelines, water disinfection and legislations to control the progression of the outbreak (**Table 6**).

**Table 6:** Enteric fever outbreak preventive measures (N = 15)

Source of outbreak	Studies supporting	Place of outbreak
**Health education (n = 7)**	Yan et al (2015) [21], Yan et al (2016) [22]	China (n = 2)
	Cherian et al (2014) [23]	India (n = 1)
	al-Zubaidy et al (1995) [24]	Saudi Arabia (n = 1)
	Jonathan et al (1999) [25]	Tajikistan (n = 1)
	Mwansa et al (2017) [26]	Zambia (n = 1)
	Imanishi M et al (2014) [27]	Zimbabwe (n = 1)
**Mass immunization (n = 4)**	Yan et al (2016) [22]	China (n = 1)
	Meltzer et al (2013) [28]	Nepal (n = 1)
	Goh et al (1992) [29]	Singapore (n = 1)
	Bodhidatta et al (1987) [30]	Thailand (n = 1)
**Water disinfection and sanitary measures (n = 7)**	Yan et al (2015) [21], Yan et al (2016) [22], Wang et al (2017)	China (n = 3)
	Cherian et al (2014) [23]	India (n = 1)
	Jonathan et al (1999) [25]	Tajikistan (n = 1)
	Mwansa et al (2017) [26]	Zambia (n = 1)
	Imanishi M et al (2014) [27]	Zimbabwe (n = 1)
**Building & construction of treatment plants and pipelines(n = 4)**	Yan et. al, (2015) [21]	China (n = 1)
	Banerjee et al (2007) [31], Cherian et al (2014) [23].	India (n = 2)
	Aye et al (2004) [32]	Myanmar (n = 1)
	Jonathan et al (1999) [25]	Tajikistan(n = 1)
**Legislation (n = 3)**	Yan et al (2016) [22], Wang et al (2017)	China (n = 2)
	Teoh et al (1997) [33]	Singapore (n = 1)
**Others (n = 1) (Cholecystectomy of convalescent carriers)**	Goh (1981) [34]	Singapore (n = 1)

## DISCUSSION

In this systematic review, we observed only 68 studies published from 1965 to 2019 were eligible for data extraction. Likewise, outbreaks of enteric fever occurred in geographical clusters, resulting in the majority of the reports from only a few large countries such as India and China. While there were no reports of enteric fever outbreaks available until 1975, in the succeeding decades a continuous increment in the number of reported outbreaks of enteric fever was observed. No standard case definitions for a line listing of suspected or confirmed cases of enteric fever, laboratory testing, unified outbreak scoring system, or a checklist was used for the identification of enteric fever outbreak. The finding of this systematic review including published literature from the last 50 years has important public health and clinical implications, calling for an urgent need for the development of a unified scoring system or checklist for the identification of enteric fever outbreaks globally. The development of such a unified outbreak scoring system or checklist will not only facilitate the early and timely identification of the outbreaks possible, but it will also make the reporting and comparison of data across different countries a reality.

In Asian countries, the outbreaks of enteric fever occurred in geographical clusters. This spatial clustering was observed during the outbreak from 1987 to 1991 in China, Pakistan, India, and Bangladesh. This depicts the risk that an outbreak reported in one geographical boundary may penetrate to a neighboring geographical area as well. Around 70% of studies demonstrated that water contamination and inappropriate sanitary measures were the identified source of transmission. This was also supported by other studies where the disease is highly endemic because of lack of sanitary measures and access to clean water [35,36]. The risk factors of enteric fever vary within the geographical boundary [35] and no TRFs (Typhoid Risk Factor stratification) has been used or developed to identify inter-countries risk factor or source of transmission.

The increase in the number of enteric fever outbreaks in each succeeding decade could be because of the increase in publication trend, reporting, and availability of online supplements. While no reports eligible for inclusion in this study were found during the first decade (1965-75), a gradually increasing number of papers published on enteric fever outbreak was found from 1976 and onwards. The changing landscape of publication tendency over time might also have resulted in the publication of only severe form of outbreaks to be reported in the past while minor or small outbreaks to be reported recently, causing some form of bias in our results. While this is possible, we did not find any difference in the level (geographic distribution) or severity of the outbreaks over the period.

The definition of a suspected or a clinical case of enteric fever was very heterogeneous among studies in different regions. In addition, enteric fever case definition varied within the same geographical region, highlighting the lack of a standardized case definition for use in outbreak settings. The non-specificity of the clinical definition of enteric fever was also highlighted in a recent WHO report [1]. Symptoms such as fever, abdominal discomfort, diarrhea, and nausea and vomiting were considered by more than 50% of studies as clinical criteria for diagnosis of enteric fever, however, the reporting was insufficient and inconsistent in various studies. Most outbreaks were due to an MDR isolate. An unexpectedly high number of MDR and FQR outbreaks were observed among the studies published after the 2000s., this might represent a publication bias because the number of studies published before the 2000s are very few and that cannot explain the high virulence or greater tendency of the spread of MDR isolates. Because of the limitation of reporting of outcomes and hospitalization in most outbreaks, disease severity and case-fatality rates in the outbreaks cannot be assessed [36,37].

### Applicability and implications for research

Enteric fever is one of the highly endemic diseases among the Asian and African countries [38,39], but unfortunately, the regional surveillance system for the reporting of enteric fever is almost non-existent among the Asian and African countries [36,37], therefore there is a dire need to establish an association that reports incidence, prevalence, hospitalization, complications, treatment outcome and mortality of enteric fever both at the community as well as at institutional level. The clinical signs and symptoms for enteric fever case diagnosis are not specific and they are often indistinguishable from other febrile illness[40]. Therefore, a thorough review of hospital records and clinical data of all suspected, probable, and confirmed cases of enteric fever can help in standardizing the case definition of enteric fever. The syndromic definition of enteric fever would result in series of positive outcomes. Like, it would aid in reducing the false positive cases of enteric fever and would also control the irrational empirical antibiotic use. Since the emergence of MDR, FQR and XDR cases are the results of irrational use of antibiotics therefore, the controlled practice of empirical antibiotic use would be a step for prevention and control of MDR, FQR and XDR cases [41].

In this systematic review we observed substantial variation in the diagnostic testing, and lack of any unified outbreak scoring or a standardized global checklist for the early identification and impact assessment of enteric fever outbreaks. An experience from the recent Ebola outbreak in West Africa provided an important lesson regarding the importance of initiating public health response early at the initial stages of an outbreak. Any delay in the early stages of an outbreak can result in an exponential increase in the scale of an outbreak, causing substantial follow-on effects [42,43]. A validated outbreak scale with the potential to predict the risk of an outbreak of a particular disease in an early stage can be beneficial in identifying outbreaks of catastrophic potential [44]. Several studies have reported the utility of outbreak scales containing various pathogen and country-specific parameters for example the novelty of the causative agent, its virulence (resistance to available antibiotics), incidence rate, fatality rate, source of transmission, availability of treatment/vaccine, population density especially susceptible population, political stability, preparedness of local health care system to deal with the outbreak, and availability of local financial, technical and trained human resources, for the timely identification of outbreaks of other infectious diseases eg, Middle East Respiratory Syndrome coronavirus (MERS-CoV), severe acute respiratory syndrome (SARS), and Ebola [44,45]. Likewise, global risk assessment tools are available for the early detection of polio, measles and dengue outbreaks which are globally used in several endemic countries as a decision support tool for the characterization of an outbreak and prioritization of response [46-48]. We recommend the development of a unified enteric fever outbreak scoring framework for early identification and prioritization of the public health response.

The spread of disease can be prevented by identifying the population at risk. Every person is at equal risk of getting the disease, but travellers are more likely to transmit the infection to others [49,50]. International travellers from Pakistan, India, Bangladesh, Mexico, Haiti, and the Philippines largely contribute to the international transmission of typhoid fever [49]. Therefore, introducing an active immunization strategy against Typhoid can aid in controlling the spread [44] of typhoid fever from the carriers to the healthy population. The typhoid carriers living in typhoid endemic countries can transmit typhoid infection in typhoid-free communities and nations very easily [51]. Different advisory bodies of the developed nations, ie, CDC of United States of America, CATMAT (Committee to Advise Tropical Medicine and Travel) of Canada and NaTHANaC (National Travel Health Network and Center) of the United Kingdom all have recommended typhoid vaccination for travellers and high-risk group people travelling to high-risk typhoid region [52-54]. Hence, immunizing the people of developing nations against typhoid vaccine could aid in preventing the geographical penetration of the disease.

An epidemiological transition has been observed among the cases of enteric fever. Children under 15 years of age are more vulnerable to enteric fever compared to adults. Vaccination against Typhoid is the sustainable solution stated by Coalition Against Typhoid (CAT) [55]. Many studies also supported typhoid vaccination for the prevention of typhoid fever [56-58]. Thus, the typhoid vaccine will provide multiple benefits not limited to outbreak prevention, but it will also reduce antimicrobial resistance and irrational empirical treatment via broad-spectrum antibiotics [59].

In future, the researchers must focus on assessing the knowledge gap and on implementing the effectiveness of different prevention strategy for reducing the disease episodes and burden. Moreover, countries must focus on water chlorination, water plant and sewerage plant infrastructure development and legislation for proper sanitation, hygiene and vaccinations among the food handlers, health care workers, international travellers and other high-risk groups of individuals and communities. Also, there is a global need of developing a unified enteric fever outbreak scores or checklist to detect outbreak at an early stage and prioritize timely response.

### Strengths and limitations

This review has certain strengths, such as the use of the breadth of databases, inclusion of original research articles reporting a single outbreak, and inclusion of last 50 years data of all typhoid, and paratyphoid outbreaks that occurred in Asia and Africa. However, the exclusion of the studies which were published in languages other than English weaken the internal validity of this review, and there are many Asian and African countries where the native language is used for scientific publication and reporting. Because of this exclusion, the literature retrieved for this study does not represent the actual outbreak events among Asian and African countries. Further, there is also a potential issue of cohort effect due to the inclusion of 50-year retrospective records eg, further back in the past you go, the less likely it is for an epidemic to be published. The cohort effect might have resulted in the bias of unknown effect and even direction, it might easily be the case that only the severe one’s outbreaks were published in the past, while even smaller get published today. The problem is a constant change in the landscape including hygiene and sanitation, making the longer-term comparison less likely to be directly comparable. Moreover, due to the heterogenicity of reporting mechanism and outcomes, quality assessment and meta-analysis of was not done.

## CONCLUSION

Our review highlights the variability in detection and reporting methods for enteric fever. Case definitions and laboratory methods for the diagnosis of enteric fever cases were not standardized. There is lack of a standardized “tool kit” for containing the outbreak if an event happens. This review highlights the necessity of the development of a unified enteric fever outbreak scale or a standardized global scoring system for the early identification of an outbreak and prioritization of public health response.
